# Encapsulation of W/O/W Acerola Emulsion by Spray Drying: Optimization, Release Kinetics, and Storage Stability

**DOI:** 10.3390/foods13101463

**Published:** 2024-05-09

**Authors:** Yen Thi Dang, Hieu Tran, Tuyen Chan Kha

**Affiliations:** 1 Faculty of Food Science and Technology, Ho Chi Minh City University of Industry and Trade, Ho Chi Minh City 700000, Vietnam; yendt@fst.edu.vn; 2 Faculty of Chemical Engineering and Food Technology, Nong Lam University, Ho Chi Minh City 700000, Vietnam; hieutran10299@gmail.com

**Keywords:** acerola, encapsulation, release kinetics, spray drying, storage stability

## Abstract

Acerola (*Malpighia emarginata* DC.) is a sub-tropical and tropical fruit renowned for its high levels of vitamin C and phenolic compounds, which offer health benefits. This study aimed to optimize the spray drying process by determining the inlet and outlet temperatures using response surface methodology (RSM) with the central composite design. Additionally, it aimed to evaluate the release kinetics in the hydrophilic food simulation environment and the stability of the resulting powder under various storage temperatures. The RSM method determined the optimal inlet and outlet temperatures as 157 °C and 91 °C, respectively. High-accuracy prediction equations (R^2^ ≥ 0.88) were developed for moisture content (3.02%), process yield (91.15%), and the encapsulation yield of total polyphenol content (61.44%), total flavonoid content (37.42%), and vitamin C (27.19%), with a predicted monolayer moisture content below 4.01%, according to the BET equation. The powder exhibited good dissolution characteristics in the acidic hydrophilic food simulation environment and showed greater stability when stored at 10 °C for 30 days, compared to storage at 35 °C and 45 °C.

## 1. Introduction

Acerola (*Malpighia emarginata* DC.) is indigenous to the Caribbean and is renowned for its numerous health benefits, including its antihyperglycemic effect [1], hepatoprotective effect [2], skin lightening [3], and its potential in preventing hyperglycemia and dyslipidemia in diabetic individuals [4]. Acerola fruits are particularly notable for their exceptionally high vitamin C content, ranging from 1500 to 4500 mg per 100 g, which is approximately 50 to 100 times higher than that of oranges or lemons [5]. Additionally, they contain significant amounts of phenolic compounds, with about 452 to 751 mg gallic acid equivalent (GAE) per 100 g frozen pulps [6]. According to the Institute of Medicine [7], the Recommended Dietary Intake (RDI) of vitamin C for women and men (aged 19 years and above) is 75 mg and 90 mg per day, respectively, with the upper limit of 2000 mg per day for all adults, roughly equivalent to consuming just two–three acerola fruit per day.

While acerola offers significant nutritional benefits, its applications in the food industry, notably in beverage production like juice, soft drinks, and liqueurs, poses certain challenges. One such challenge is the limited shelf life of mature acerola fruits, lasting merely 2–3 days at ambient temperature [8]. Moreover, heat-sensitive components in acerola juice can be compromised during processing, which may adversely affect the quality and appeal of the final food product.

Emulsification technology offers promising solutions to extend the shelf life and preserve bioactive compounds in acerola juices. Emulsion-based delivery systems, particularly double W/O/W (water-in-oil-in-water) emulsions, can effectively encapsulate various bioactive compounds, such as vitamins, polyphenols, and antioxidants [9]. These multilayer structures not only enhance the bioavailability, taste, and texture of the bioactive compounds but also provide protection against environmental factors, such as light, oxygen, and heat, thereby maintaining their stability and integrity during storage and processing [10]. In addition, emulsions allow for the customization of release kinetics, enabling sustained effects or targeted delivery by adjusting the emulsion compositions [11].

Along with emulsification, the spray drying process, particularly the control of inlet and outlet temperatures, plays pivotal roles in the efficacy of the encapsulation [12,13]. This process aims to improve the delivery of bioactive compounds into foods while enhancing their stability, bioavailability, and functionality during processing and storage, thus minimizing undesirable interactions with the food matrix [14,15]. Despite the elevated temperatures employed, spray drying minimally affects the chemical composition of these compounds and is significantly more cost-effective than spray chilling encapsulation [16,17]. For instance, Bringas-Lantigua et al. [17] successfully applied spray drying to microencapsulate yellow lemon essential oil, achieving a high retention rate of volatile oils, reaching 95.7%, and microencapsulation efficiency up to 99.9%. Additionally, spray drying allows for precise control over particle size and morphology, making it a preferred option for large-scale production. However, it is essential to optimize the inlet and outlet temperatures to ensure the highest quality of encapsulated powder, as excessively high or low temperatures can adversely affect its physical and chemical properties [12,13,18].

The temperatures utilized in spray drying exert a considerable influence on encapsulation efficiency [19]. Moreira et al. [20] proved that the degree of ascorbic acid in acerola pomace spray-dried powder was impaired by increasing the inlet temperature from 170–200 °C. Hence, it is crucial to meticulously control and optimize drying temperatures to achieve efficient spray drying while upholding desired outcomes concerning encapsulation yield and product characteristics. Furthermore, it is imperative to explore if the properties of the powder are impacted by storage temperature and relative humidity. This investigation will enable the determination of suitable storage conditions. Additionally, the release kinetics of the encapsulated powder in a food simulant environment should be assessed, to ensure the controlled and efficient release of encapsulated compounds for intended applications.

Despite the importance of encapsulation techniques in preserving bioactive compounds, there is currently limited research on the encapsulation of these compounds in acerola fruit using the spray drying, especially with W/O/W emulsion. This study aimed to address this research gap by optimizing the inlet and outlet spray drying temperatures for encapsulating acerola fruit W/O/W emulsion. Additionally, the study sought to assess the stability of the optimized powder under various storage temperatures and conduct release assays in an aqueous food simulation. Parameters considered for optimization included process yield, encapsulation yield of bioactive compounds (e.g., vitamin C, total phenolic content, and total flavonoid content), moisture content, bulk density, DPPH-free radical scavenging ability, and particle morphology.

## 2. Materials and Methods

### 2.1. Chemicals and Ingredients

Methanol (CAS 67-56-1, 99.9%), sodium hydroxide (NaOH, CAS 1310-73-2, ≥97%), o-phosphoric acid (H_3_PO_4_, ≥85%) and Tween 80 (CAS 9005-65-6, extra pure) were sourced from Fisher Scientific (Waltham, MA, USA). Sodium carbonate (Na_2_CO_3_, CAS 497-19-8), sodium nitrite (NaNO_2_, CAS 7632-00-0), and potassium carbonate (K_2_CO_3_, CAS: 584-08-7) were obtained from Scharlau (Barcelona, Spain). Aluminum chloride (AlCl_3_, CAS 7784-13-6, >98%), sodium chloride (NaCl, CAS 7647-14-5, >99%), and potassium chloride (KCl, CAS 7447-40-7, >99%) was sourced from Duchefa Biochemie (Haarlem, The Netherlands). 2,2-Diphenyl-1-picrylhydrazyl (DPPH, CAS 1898-66-4), gallic acid monohydrate (CAS 5995-86-8, ≥99), L-ascorbic acid (CAS 50-81-7, 99%), quercetin (CAS 117-39-5, ≥95%), catechin (CAS 18829-70-4, ≥97%), and (-)-epicatechin (CAS 490-46-0, ≥98%) from Sigma Aldrich (Saint Louis, MO, USA). Acetic acid glacial (CAS 64-19-7, 100%) and acetonnitrile for HPLC (CAS 75-05-8, ≥99.9%) from Merck (Rahway, NJ, USA). Folin and ciocalteu’s phenol reagent (RM10822) from Himedia (Mumbai, Maharashtra, India), potassium acetate (CH_3_COOK, Cas 127-08-2, >92%) from Xilong (Guanzhou, China), polyglycerol polyricinoleate (Finamul-VR-42, INS 476) was obtained from Fine Organics Industries (Mumbai, Maharashtra, India), and refined sunflower oil from Tuong An Vegetable Oil Jsc. (Hanoi, Vietnam).

Gum Arabic (GA, INS 414) and maltodextrin (MD, DE 15-20) purchased from Qinhuangdao Lihua Starch (Qinhuangdao, China) were used as wall materials.

### 2.2. Plant Material, Aqueous Extraction and Concentration

Upon procurement from the Go Cong Dong Co-operative (Tien Giang Province, Vietnam), fresh acerola underwent a gentle washing process with water to eliminate visible dirt. Subsequently, it was immersed in a 100-ppm chlorine solution for 5 min to eradicate surface microorganisms. Following this, the acerola was rinsed, drained, and subjected to juicing twice using the HSJ-B30A slow juicer (Hafele, Nagold, Germany). The extracted juice was then filtered through cheesecloth to eliminate insoluble residues before being prepared for cryoconcentration.

The concentration process was conducted utilizing the cryoconcentration method [21]. In this process, each batch of juice underwent freezing in three stages at −18 °C for 24 h, 36 h, and 60 h, respectively. After each freezing stage, the juice was thawed, until 50% of the initial volume was obtained to proceed to the next concentration cycle, while the remaining ice was removed. Thawing was facilitated with microwave assistance (at a power of 560 W), and the concentrated juice was stored at −18 °C for subsequent experimentation.

### 2.3. W/O/W Emulsion Preparation

The W/O/W emulsion was prepared in two sequential steps according to our optimization results. Initially, a W/O emulsion was formed by stirring concentrated acerola juice (40%, *v*/*v*) with an oil phase (60%, *v*/*v*) comprising PGPR (Polyglycerol polyricinoleate, 10%, *v*/*v*), using the HSC-7 magnetic stirrer (VELP Scientifica, Usmate Velate, Italy) at 700 rpm for 20 min. In this step, the oil served as the continuous phase while the PGPR acted as a hydrophobic emulsifier, reducing the surface tension and dispersing the juice in the oil phase. This occurred because the hydrophilic end of the emulsifier molecule was attracted by the aqueous phase, while its hydrophobic end was attracted to the oil molecules. This molecular arrangement helped the oil phase form a protective barrier around the water droplets, preventing them from merging and maintaining the stability of the emulsion.

Subsequently, this emulsion (21%, *v*/*v*) was incrementally added dropwise into a secondary water phase containing Tween 80 (25%, *v*/*v*) and stirred at 900 rpm for 30 min to achieve the formation of the W/O/W emulsion. At this point, Tween 80, a hydrophilic non-ionic emulsifier, positioned its hydrophilic heads towards the water droplets, while the hydrophobic tails extended outward into the oil phase of the W/O emulsion. By aligning at the interface between the oil and water phases, Tween 80 molecules reduced the surface tension between the two phases, dispersed W/O droplets into the second water phase, and formed a protective film around the W/O droplets. This action helped prevent droplets from coalescing, thereby stabilizing the emulsion and maintaining its structure over time. This W/O/W emulsion was utilized as the core material in the spray drying process.

### 2.4. Infeed Solution Preparation

Based on our preliminary studies, a blend of MD and GA in a ratio of 2:1 (*w*/*w*) was chosen as the wall material for encapsulation because of its ability to reduce the viscosity and adhesion of the infeed solution, thus enhancing spray drying efficiency and powder stability [22]. Initially, the encapsulation wall stock solution (30%, *w*/*w*) was prepared by dissolving the MD and GA mixture in deionized water, followed by overnight activation. Subsequently, the W/O/W emulsion was combined with this stock solution in a ratio of 1:1.5 (*w*/*w*) and homogenized using the SHG-15A homogenizer (SciLab, Seoul, Korea) at 4000 rpm for 10 min to facilitate the encapsulation process. The composition of the infeed solution included the following components: MD (12.0%), Tween 80 (10.0%), GA (6.0%), refined sunflower oil (2.5%), concentrated juice (2.1%), and PGPR (0.2%). These percentages were adjusted to account for dried weight, resulting in the following equivalents: MD (36.6%), Tween 80 (30.5%), GA (18.3%), refined sunflower oil (7.6%), concentrated juice (6.4%), and PGPR (0.6%).

### 2.5. Response Surface Model

The optimal inlet (X_1_) and outlet (X_2_) temperatures for the spray drying process were determined using response surface methodology (RSM). The 2^3^ Central Composite Design (CCD) was employed to develop a second order model for predicting the dependent variables, which included process yield, encapsulation yield of TPC, TFC, and Vitamin C, moisture content, and DPPH-free radical scavenging ability. The two independent variables were examined at five levels, as shown in Table 1. 

The experimental data were fitted to Equation (1) as follows:

Y_i_ = a_o_ + a_1_X_1_ + a_2_X_2_ + a_11_X_1_^2^ + a_22_X_2_^2^ + a_12_X_1_X_2_ (1)

In the regression mode, Y_i_ represents the dependent variable; X_1_ and X_2_ denote the levels of the independent variables for the linear, quadratic, and interaction terms, respectively; while a_o_, a_1_, a_2_, a_11_, a_22_, and a_12_ are coefficients to be determined.

### 2.6. Spray Drying

The infeed solution was dried using a laboratory-scale spray dryer (SD-06, Lab Plant, North Yorkshire, UK). The airflow rate was maintained at 30 m^3^/ h, while the inlet drying temperature was directly set according to the model provided in Table 1. The outlet temperature was controlled using the feed flow rate. Following drying, the powder was allowed to cool, weighed, and subsequently sealed in flat laminated aluminum bags for storage at −18 °C until further use. 

### 2.7. Release Kinetics

In vitro release experiments were performed on the optimal encapsulated powder using three different media: water, 10% ethanol, and 3% acetic acid, designated as food simulants for aqueous and acidic food products, respectively, according to Commission Regulation 10/2011 EU (10/2011/EC). The dried powder was dissolved in each medium at a ratio of 1:1 (*w*/*v*) at ambient temperature. At appropriate time intervals, the dispersions were centrifuged at 4000 rpm for 5 min to collect an aliquot of the supernatant for analysis. The released content of each index was calculated using the following formula:

$$Released\;content\left(\%\right)=\frac{Total\;bioactive\;content\;in\;supernatant}{Total\;bioactive\;content\;in\;powder}\times 100$$

To characterize the appropriate release kinetic profile, three food model approaches were considered: Korsmeyer–Peppas, Higuchi, and Peppas–Sahlin empirical models.

- Korsmeyer–Peppas equation: Q_t_ = K_M_ × t^n^
- Peppas–Sahlin equation: Q_t_ = K_1_ × t^n^ + K_2_ × t^2n^
- Higuchi equation: Q_t_ = K_H_√t

Here, Q_t_ represents the percentage of TPC, TFC, and vitamin C (%) and DPPH (%) released at time t, compared to their initial contents; and K_M_, K_1_, K_2_, and K_H_ are the release constants for compounds of the Korsmeyer–Peppas, Peppas–Sahlin, and Higuchi equations, respectively. The optimal kinetic model and release mechanism are chosen based on the determination coefficient R^2^ and release exponent n, respectively.

### 2.8. Storage Stability

The stability of optimal encapsulated acerola powder was assessed under various storage temperatures. Initially, 10 g of powder was packed into individual flat laminated aluminum bags, sealed, and then stored at 10, 35, and 45 °C for a duration of 30 days. Changes in TPC, TFC and vitamin C contents were monitored on Day 0, 1, 2, 5, 10, 20, and 30. 

The degradation of TPC, TFC and Vitamin C contents in the encapsulated powders was calculated by using the standard equation for a first order kinetic model:

lnC = lnC_o_ − k(t)

The half-life (t_1/2_) was computed at a specific temperature using the equation:

t_1/2_ = ln2/k

The activation energy (E_a_) was determined utilizing the Arrhenius equation:

$$k=A\times e^{\frac{-E_{a}}{RT}}$$

where C denotes the concentration at time t; C_o_ signifies the concentration at the initial time; k is the degradation rate constant (day^−1^), derived from the slope of a plot of the natural log of C/C_o_ against time; t represents the storage duration (days); R is the gas constant (8.3145 J/K mol); T indicates the temperature in Kelvin; A (L/mol/s) is the frequency factor, considering the frequency of reactions and probability of correct molecular orientation.

### 2.9. Effect of Humidity Conditions

A total of five hermetic glass desiccators containing various saturated salt solutions were prepared to establish five different relative humidities at 25 °C. These solutions included sodium hydroxide (NaOH), potassium acetate (CH_3_COOK), potassium carbonate (K_2_CO_3_), sodium chloride (NaCl), and potassium chloride (KCl). Approximately 2 g samples of the optimal encapsulated powder were, in triplicate, placed in petri plates within each of the five desiccators and stored at 25 °C for a minimum of 20 days. Equilibrium moisture content (EMC) was reached when the sample weight remained constant across two consecutive measurements. Subsequently, the samples underwent a 24-h oven drying process at 105 °C to determine their ultimate moisture content. The monolayer moisture content (M_o_) was determined using the Brunauer–Emmett–Teller (BET) method:

$$M_{o}=\frac{MC\times C\times Aw}{\left(1-Aw\right)[1+\left(C-1\right)Aw]}$$

where M_o_ represents the equilibrium amount of water adsorbed in a single layer per 100 g of dry matter, MC denotes the moisture content of the powder, C stands for the BET constant, and Aw indicates the water activity of the powder at the given moisture content (MC). After equilibration at the five relative humidities, the DPPH scavenging capacity and the encapsulation yield of TPC, TFC, and vitamin C in the encapsulated powders were determined.

### 2.10. Analytical Methods

#### 2.10.1. Process Yield

The process yield was calculated by dividing the dry weight of the encapsulated powder obtained by the dry weight of the infeed solution, and then expressing this ratio as a percentage (%).

#### 2.10.2. The Encapsulation Yield (EY)

The EY of bioactive compounds was determined, with respect to the content of TPC, TFC, and Vitamin C (mg/g d.w.) in the powder after encapsulation and in the parent infeed solution before encapsulation, as follows:

$$EY(\%)=\frac{Bioactive\;concentration\;in\;encapsulated\;powder}{Bioactive\;concentration\;in\;infeed\;solution}\times 100$$

#### 2.10.3. Total Phenolic Content (TPC)

For sample preparation, 5 g of dried powder was dissolved into 20 mL distilled water, and it was then diluted to 10 times with distilled water. After, this, solution was centrifuged at 4000 rpm for 5 min to obtain the supernatant. The determination of TPC was carried out according to the Folin–Ciocalteu method with slight modifications [23]. Briefly, 0.5 mL of 10% Folin–Ciocalteu reagent was added to 0.5 mL of the sample. After 5 min, 2.5 mL of 20% Na_2_CO_3_ was added to the mixture, which was then shaken well. Subsequently, the sample was incubated for 1 h in the dark at ambient temperature. The absorbance of the sample was measured at 765 nm using the Agilent 8453 UV-Vis spectrophotometer (Agilent Technologies, Inc., Santa Clara, CA, USA). The TPC was calculated using a standard curve prepared from an aqueous gallic acid solution (ranging from 0.1 to 1.0 μg/mL), and the results were expressed as milligrams of gallic acid equivalents (GAE) per gram sample (mg GAE/g d.w.).

#### 2.10.4. Determination of Individual Phenolic Compounds

The concentrations of individual phenolic compounds in the encapsulated powder were determined using the HPLC method described by Chuanphongpanich and Phanichphant [24], with minor modifications. After preparation, the analysis sample was separated using an HPLC system equipped with an Agilent Eclipse XDB-C18 column (150 × 4.6 mm, 5 µm). The mobile phase consisted of acetonitrile, acetic acid adjusted to pH 3.0, and methanol. The mobile phase system was prepared and loaded into the HPLC equipment to equilibrate the columns for the subsequent steps. A 0.005 g portion of the sample was mixed with 50 mL of methanol, vigorously shaken, and then filtered using a 0.45 μm PTFE syringe filter. The filtered samples were transferred to HPLC vials and placed in the HPLC system tray for analysis.

#### 2.10.5. Total Flavonoid Content (TFC)

The total flavonoid content was measured using the method of Wu and Ng [25], with slight modifications. Initially, 5 g of dried powder was dissolved in 20 mL of water, then centrifuged at 4000 rpm for 5 min to obtain the supernatant. Subsequently, 1 mL of the supernatant was mixed with 0.3 mL of 5% (*w*/*v*) NaNO_2_ solution in 4 mL distilled water. After 5 min, 0.3 mL of 10% (*w*/*v*) AlCl_3_ solution was added. Following an additional 10 min, 2 mL of 1M NaOH was introduced, and the mixture was then brought to a final volume of 10 mL with distilled water. The absorbance of the resulting solution was measured at 510 nm using the Agilent 8453 UV-Vis spectrophotometer. TFC content was determined using a standard curve constructed from quercetin solutions ranging from 0.1 to 1 μg/mL, and the results were expressed as milligrams of quercetin equivalents (QE) per gram of sample (mg QE/g d.w.).

#### 2.10.6. Vitamin C Content

The Vitamin C content in W/O/W emulsions and encapsulated powders was determined using the HPLC method [26]. After preparation, the samples were separated using an Agilent 1200 HPLC (Santa Clara, CA, USA) equipped with an Agilent Eclipse XDB-C18 column (150 × 4.6 mm, 5 µm). Approximately 900 mL of deionized water were poured into a 1000 mL volumetric flask, followed by the addition of 203 μL of 85% H_3_PO_4_. Sufficient water was then added to reach a final volume of 1000 mL. Afterwards, the solution was sonicated and degassed for 10 min before being filtered by using a membrane filter with a pore size of 0.2–0.45 μm. Next, 1.25 mL of sample was mixed with 3% meta-phosphoric acid (HPO_3_) until the total mixture volume reached 1 L [6]. A sample volume of 20 μL was injected into the HPLC system at a flow rate of 0.5 mL/min, with the column temperature maintained at 40 °C. Detection was carried out using a DAD detector (G1315D) set at 270 nm. The identification of ascorbic acid was based on the retention time of a peak, compared with the authentic standard.

#### 2.10.7. Antioxidant Activity

The antioxidant activity was assessed utilizing the DPPH-free radical method, as described by Brand–Williams [27], with certain modifications. The sample solution and the DPPH powder was dissolved in 80% methanol. Subsequently, 3 mL of 0.1 mM DPPH solution was added to 150 µL of the diluted sample. The mixture was thoroughly shaken and then incubated in the dark for 30 min. Finally, the absorbance of the sample was measured at 517 nm using a UV-Vis spectrophotometer, and the DPPH-free radical scavenging (%) was determined using the following formula:

$$DPPH\;free\;radical\;scavenging\;(\%)=\frac{A_{o}-A}{A_{o}}\times 100$$

where A_o_ represents the absorbance of the control sample, and A denotes the absorbance of the sample.

#### 2.10.8. Moisture Content (MC)

The moisture content of the samples was analyzed gravimetrically using an AnD MX-50 moisture analyzer (A&D Company, Ltd., Tokyo, Japan). A 2 g sample was placed on the sample pan, and the standard mode with a drying temperature set to 150 °C for analysis was then used. The analyzer was operated under ambient temperature.

#### 2.10.9. Bulk Density

The bulk density was measured by allowing a 2 g sample to freely settle into a 50 mL cylinder, then dividing the weight by the recorded volume (g/mL).

#### 2.10.10. Powder Particle Morphology

Particle morphology was assessed using a scanning electron microscope (SEM) (JSM-IT200, JEOL, Japan). A small amount of each sample was affixed to the sample holder using double-sided tape. The sample holder was then subjected to vacuum conditions in an SPI gold spray coater, coating the powder with a fine layer of gold palladium. Subsequently, samples were examined and photographed at magnifications of 100×, 250×, 1000×, and 2500×.

#### 2.10.11. X-ray Diffraction Analysis (XRD)

An amount of encapsulated powder sample was placed into the sample slot and then pressed with frosted glass to create a suitable surface texture suitable for analysis. XRD patterns were recorded on a 2θ scale from 10 to 80°, with an increment of 0.02° and a fixed lifetime of 0.5 s, on an X’Pert PRO Powder Diffractometer (Malvern Panalytical Ltd., Worcestershire, UK).

### 2.11. Statistical Analysis

All experiments and corresponding analyses were conducted in triplicate. A JMP software version 13.0 (SAS Institute Inc., Cary, NC, USA) was used to generate three-dimensional (3D) surface, two-dimensional (2D) contour, and prediction profiler plots for process yield, MC, encapsulation yield of TPC (EY_TPC_), TFC (EY_TFC_), and Vitamin C (EY_vitamin C_). The suitability of the second order polynomial model was evaluated using the coefficient of determination (R^2^) and the lack of fit. Significant differences between means at *p* < 0.05 were determined using the SPSS software version 23.0 statistical package (IBM Australia Limited, St Leonard, NSW, Australia). Pearson correlation coefficients were also calculated using the SPSS software.

## 3. Results and Discussion

### 3.1. Response Surface Optimization

#### 3.1.1. Fitting the Response Surface Models

The RSM with CCD was employed to investigate the optimal inlet (X_1_) and outlet (X_2_) temperatures. The statistical analysis showed that neither temperature significantly influenced the bulk density (Table 2). Consequently, the influence of inlet and outlet temperatures on bulk density was not taken into consideration to evaluate the optimal results of these two temperatures.

Encapsulated powder parameters including MC, process yield, EY_TPC_, EY_TFC_, and EY_vitamin C_ were selected to participate in the optimization model. Table 3 illustrates the experimental data (Exp.) and the predictions (Pred.) made using the optimal equations, as follows:
                      MC (%) = 76.62 − 0.01X_1_ − 1.51X_2_ − 0.002X_1_X_2_ + 0.0005X_1_^2^ + 0.01X_2_^2^
                      Process yield (%) = −5683.59 + 57.31X_1_ + 28.13X_2_ − 0.08X_1_X_2_ − 0.16X_1_^2^ − 0.09X_2_^2^                      EY_TPC_ (%) = −8594.64 + 95.31X_1_ + 26.72X_2_ + 0.05X_1_X_2_ − 0.31X_1_^2^ − 0.19X_2_^2^                      EY_TFC_ (%) = −5046.48 + 66.14X_1_ − 1.67X_2_ + 0.11X_1_X_2_ − 0.24X_1_^2^ − 0.08X_2_^2^                      EY_vitamin C_ (%) = −2853.78 + 33.12X_1_ + 6.85X_2_ + 0.02X_1_X_2_ − 0.11X_1_^2^ − 0.05X_2_^2^


The data analysis presented in Table 3 indicates that the parameters, such as moisture content, process yield, EY_TPC_, EY_TFC_, and EY_vitamin C,_ obtained from experiments were similar to calculated results from the optimal prediction equation (R^2^ ≥ 0.88). Additionally, the lack of fit test results (Table 4) indicated the influence of input temperature and outlet temperature during the drying process on the quality of powder products was found to be insignificant (*p* > 0.05). Table 4 also shows that the inlet temperature had both significant linear and quadratic effects on the process yield, EY_TPC_, and EY_TFC_, while only having a linear effect on the MC and a quadratic effect on the EY_vitamin C_. In addition, the outlet temperature had a significant linear and quadratic effect on the MC, and had significant quadratic effect on the remaining investigated parameters, except for process yield. The only significant interaction effect of the inlet and outlet temperatures was on the EY_TFC_.

**Figure 1:**
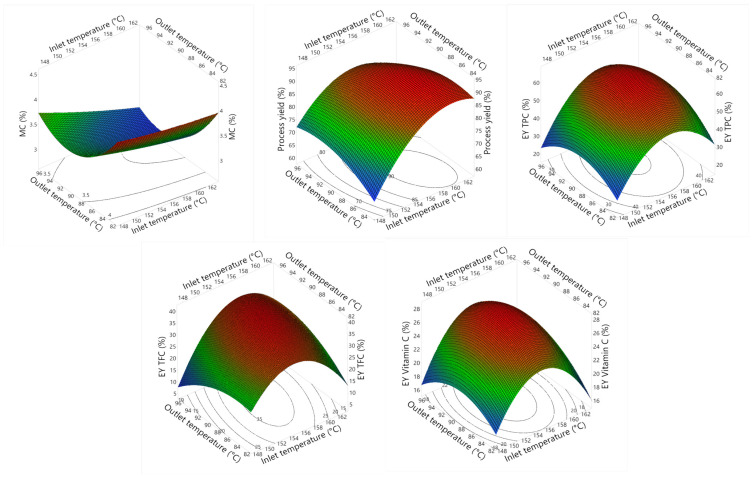
The 3D response and 2D contour plots of MC, process yield, EY_TPC_, EY_TFC_, and EY_vitamin C_ affected by inlet (X_1_) and outlet (X_2_) temperatures.

**Figure 2:**
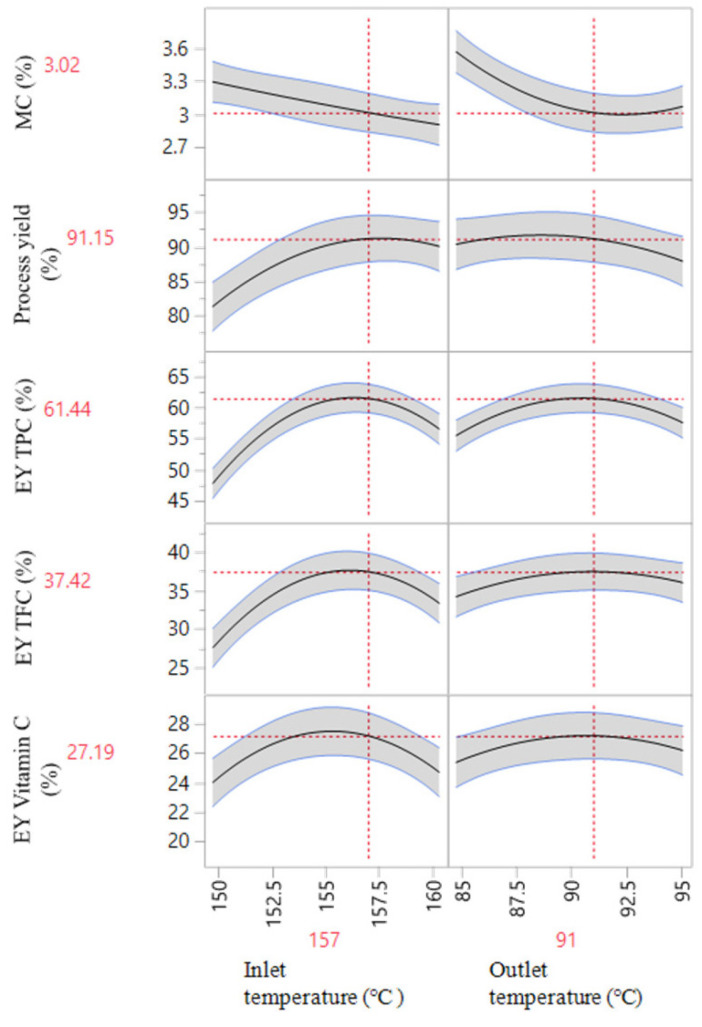
Prediction profilers of MC, process yield, EY_TPC_, EY_TFC_, and EY_vitamin C_ as a function of inlet (X_1_) and outlet (X_2_) temperatures.

Based on the effects of X_1_ and X_2_, Figure 1 and Figure 2 illustrate a 3D response surface model with a 2D contour plot and profilers predicting MC, process yield, EY_TPC_, EY_TFC_, and EY_vitamin C_, respectively. In general, as the inlet temperature increased, the MC tended to decrease, while the process yields increased. Additionally, the three remaining parameters saw a significant rise when the temperature increased from 150 °C to about 158 °C, then declined. The lower process yield and EY at low drying temperatures could be explained by the insufficient heat to dry powders, causing wall sticking and the decomposition of active compounds. In contrast, high drying temperatures could also lead to this phenomenon because the particle temperature increases faster than the glass transition temperature, leading to adhesion and reduced powder encapsulation yield [28].

When the outlet temperature increased from 82 °C to about 90 °C, the MC and EY_TFC_ tended to decrease, while the other responses increased significantly, followed by a contrasting trend. This phenomenon can be explained by the fact that increasing outlet drying temperature would lead to overheating of the particles [29]. At this time, water quickly evaporates, reducing humidity, and destroying wall layers and bioactive compounds inside the core, due to direct contact with high-temperature conditions.

Several studies in the field of encapsulation have shown similar trends. The research conducted by Corrêa-Filho, Lourenço, Moldão-Martins and Alves [30] on β-Carotene microencapsulation using GA in the inlet temperature range of 110–200 °C showed that drying yield is affected by a quadratic effect of inlet temperature and tends to increase to reach a peak at 155 °C and then decreases sharply. Additionally, Chong and Wong [31] demonstrated that 180 °C maximized the drying yield (57%) when producing sapodilla puree by spray drying using different concentrations of maltodextrin (10–50% *w*/*v*).

As discussed previously, the five response variables, including MC, process yield, EY_TPC_, EY_TFC_, and EY_vitamin C_, were effectively fitted into their respective response surface models. Achieving an optimal product involves fine-tuning the encapsulated powder, with a primary focus on maximizing process yield, EY_TPC_, EY_TFC_, and EY_vitamin C_, while minimizing moisture content. A graphical optimization (Figure 2) suggests that an input temperature of 157 °C and an outlet temperature of 91 °C (identified by the intersection of two dotted lines) were the optimal spray drying temperatures, with a desirability of 92.6%, to create high-quality encapsulated acerola powder. Generally, at the optimal spray drying inlet and outlet temperatures, as the droplets continue to lose moisture, the viscosity of the liquid carrier increases, forming a shell around the bioactive compound. This shell acts as a protective barrier, encapsulating the bioactive compound within. In this study, the bioactive compounds were protected by double layers and then spray-dried to form a protective shell, thus safeguarding the bioactive compounds during storage.

#### 3.1.2. Validation

The validation process involved spray drying samples in triplicate under optimal temperatures, comparing experimental data with predicted values. Additionally, HPLC analysis quantified key bioactive compounds. The close agreement between experimental and calculated results using prediction equations from Table 5 indicates the effectiveness of the RSM with CCD optimization model in simulating the relationship between inlet and outlet temperatures and the investigated responses. Table 6 presents the HPLC analysis results, determining the contents of Vitamin C, gallic acid, catechin, and epicatechin per 100 g dry weight of the optimal powder.

**Figure 3:**
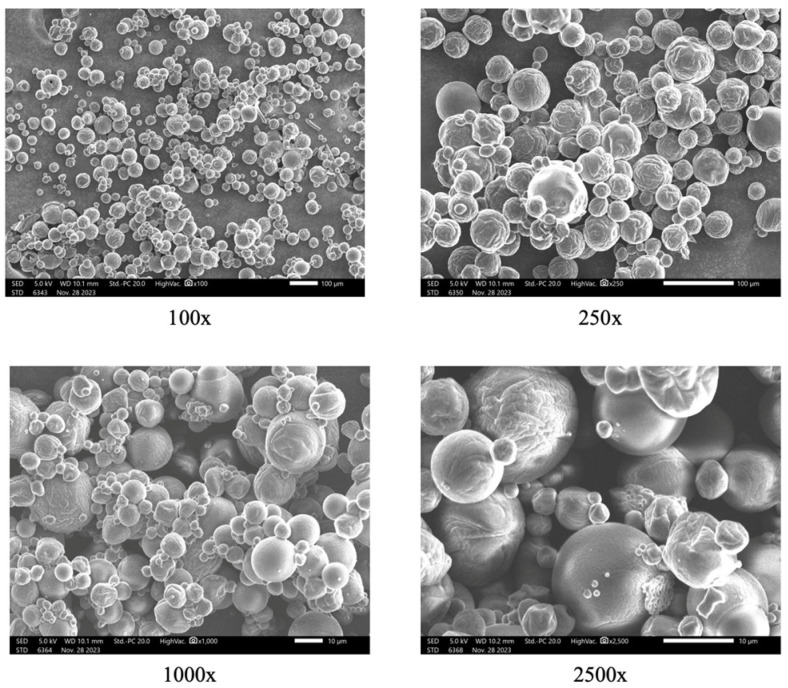
Powder particles’ morphology of encapsulated powder under optimal inlet and outlet temperatures at different magnifications.

**Figure 4:**
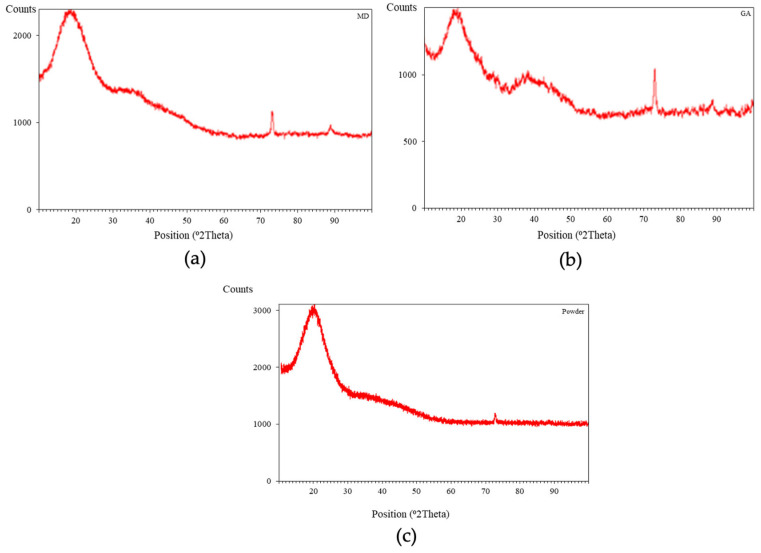
XRD analysis chart of samples: (**a**) MD, (**b**) GA, (**c**) Encapsulated powder.

The optimal powder underwent morphological analysis using SEM (Figure 3) at different magnifications, revealing a satisfactory morphological structure characterized by high uniformity and the absence of wall cracking. X-ray diffraction (XRD) analysis (Figure 4) depicted the crystal structures of the two coating components, MD and GA, as well as the microencapsulated acerola powder obtained under optimal spray drying conditions. Distinct peaks at approximately 20°, 73°, and 89° along the 2θ axis in Figure 4a indicates the crystal structure of the MD envelope, while Figure 4b displays prominent peaks at approximately 20°, 39°, 73°, and 88°, demonstrating the crystal structure of the GA shell. However, Figure 4c shows the disappearance of prominent peaks around 40° and 80–90°, with the peak near 70° shrinking in area in the microencapsulated powder analysis chart, suggesting an amorphous main structure. This amorphous structure likely resulted from interactions between the core and wall materials, as well as rapid evaporation of small molecular weight particles during the spray drying process [32]. The amorphous structure enhances solubility compared to a crystalline structure due to weaker intermolecular forces, facilitating higher bioavailability. Further studies should be conducted to evaluate the bioavailability of the encapsulated powder to clarify this. In this study, the release kinetics of the encapsulated powder and its stability are presented in the following sections.

Based on the analysis results, the optimal temperatures for the spray drying process of encapsulated acerola powder are 157 °C for inlet and 91 °C for outlet temperatures. These temperatures are anticipated to facilitate the production of powder particles with good solubility, high bioavailability, and high encapsulation yield of Vitamin C and other biologically active compounds. Therefore, it can be concluded that the adequacy of the corresponding response surface model for predicting moisture content, process yield, EY_TPC_, EY_TFC_, and EY_vitamin C_ as a function of the inlet and outlet temperatures within the experimental ranges has been confirmed. Furthermore, the high quality, characterized by the high content of bioactive compounds, of the encapsulated acerola W/O/W emulsion powder could be leveraged as a nutrient supplement in the food and pharmaceutical industries.

### 3.2. Release Kinetics

**Figure 5:**
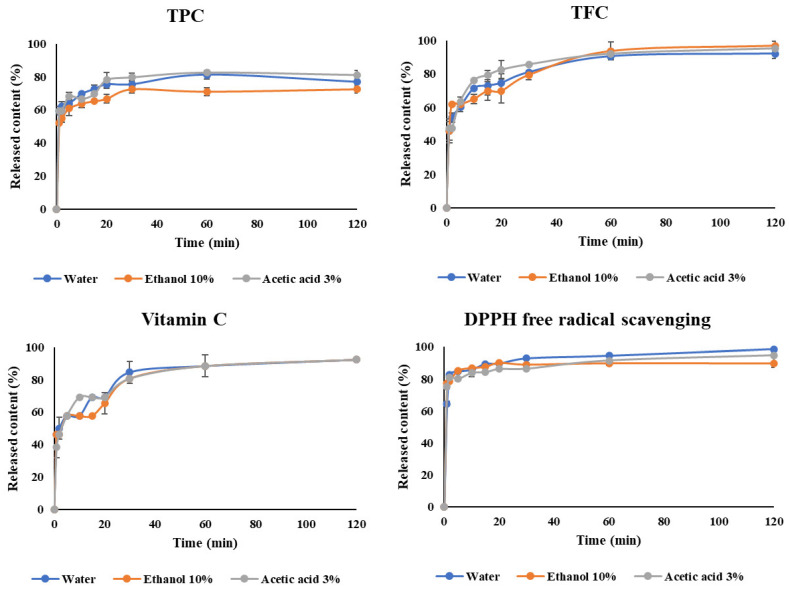
The release profile of bioactive compounds in water, ethanol 10%, and acetic acid 3%.

Understanding release kinetics is crucial for elucidating the mechanisms underlying the release of bioactive compounds, facilitating the prediction of controlled or sustained release in various systems [5,33]. Mathematical modeling offers essential tools for analyzing release data and assessing the impact of encapsulation formulation and design factors on the release mechanism. In this study, the experiment investigated the release kinetics of bioactive compounds in encapsulated powder in three different dispersion media, namely water, 10% ethanol, and 3% acetic acid, as depicted in Figure 5. It is evident that phenolic compounds and Vitamin C were quickly released during the initial 10 min of exposure to the solvents. This can be explained by the bioactive compounds being distributed mainly near the wall layer, meaning they are released quickly when the wall swells due to exposure to the dispersion medium. 

The release pattern of bioactive compounds in all three hydrophilic liquid food simulation media aligns with the typical characteristics of bioactive compound release from the matrix [34]. During the dispersion phase, the biologically active compounds tended to exhibit robust release in acidic environments and less so in ethanol environments. This can be attributed to the composition of the encapsulation wall, comprising MD and GA, which are highly polar substances with good solubility in polar solvents like water or acids. In contrast, while ethanol is a polar solvent, the presence of the ethyl group diminishes its polarity, thereby reducing the solubility of the wall in ethanol. 

The study utilized various models, including zero order, first order, Korsmeyer–Peppas, Peppas–Sahlin, Higuchi, and Weibull, to ascertain the release mechanism of biactive compounds, such as Vitamin C, TPC and TFC, as well as antioxidant activity, in three distinct environments. The findings revealed that three compatible models (Korsmeyer–Peppas, Peppas–Sahlin, and Higuchi) were employed to elucidate the release mechanisms of compounds in the food environment (refer to Table 7). The Korsmeyer–Peppas model predicted the release mechanism of biologically active compounds based on the n value. When the kinetic equation coefficient n is less than 0.45, the release mechanism follows diffusion according to Fick’s law, while a swelling-controlled system occurs when n is greater than 0.89. If the value of n falls between 0.45 and 0.89, both diffusion and swelling contribute to the release mechanism [35]. The microencapsulated powder exhibited release in all three environments through the diffusion mechanism, following Fick’s law. Additionally, the Peppas–Sahlin model indicated that, under most conditions, the microencapsulated powder is released via the diffusion mechanism, owing to the K_1_/K_2_ ratio being greater than one. However, the release of TPC and TFC from microencapsulated powder in a water environment, and TFC in an acetic acid environment, is predicted to occur through the erosion mechanism, as evidenced by a K_1_/K_2_ coefficient less than one. Based on correlation coefficients, the Korsmeyer–Peppas model is deemed appropriate for describing the release kinetics of bioactive compounds in encapsulated acerola powder (R^2^ > 0.98).

### 3.3. Storage Stability of Encapsulated Powder

**Figure 6:**
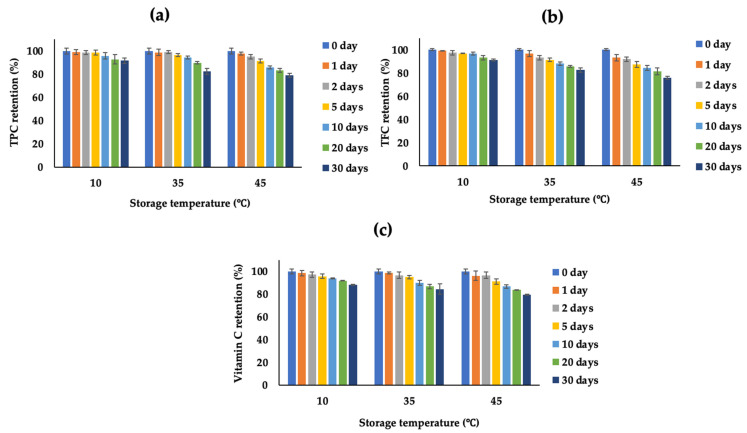
Effect of storage temperatures on the retention of TPC (**a**), TFC (**b**), and Vitamin C (**c**).

Statistical results indicated that the storage conditions significantly impacted the content of bioactive compounds and the antioxidant activity of the optimal encapsulated powder. The TPC, TFC, and Vitamin C contents tended to decrease significantly with increasing storage temperature and time. Figure 6 illustrates that the encapsulated powder remained relatively stable at 10 °C. At this temperature, there was no statistically significant change (*p* > 0.05) in quality during the first 10 days. Subsequently, the content of TPC and TFC decreased by 8–9%, while the Vitamin C content decreased by approximately 12% after 30 days. At 35 °C, the quality of the powder remained stable for the first 5 days, after which it declined by about 16–18% from the 10th day onward. Conversely, powder stored at 45 °C exhibited a faster decline in quality, with indicators decreasing by about 21–24% after 30 days of storage. These results align with several studies, indicating that the Vitamin C content in the powder decomposes more rapidly at 40 °C (91.3%), compared to 30 °C (77.8%), after 30 days of storage [36]. Additionally, the TPC and TFC in matcha (*Camellia sinensis*) decrease more rapidly when stored at 45 °C, compared to 35 °C and 25 °C.

The decrease in bioactive compounds can be attributed to oxidation and the influence of temperature. Dib Taxi et al. [37] and Mara Righetto and Maria Netto [38] have reported that using maltodextrin as a wall material efficiently conserves Vitamin C during the drying process, indicating its efficiency in the encapsulation of acerola W/O/W emulsion. Elevated temperatures accelerate the degradation rate of the encapsulation walls, resulting in the loss of protection for the core and direct exposure to external factors, such as drying temperature and humidity. This exposure leads to oxidation, changes in bioavailability [39], or structural damage [40], consequently reducing the content of biactive compounds in the powder after drying.

**Figure 7:**
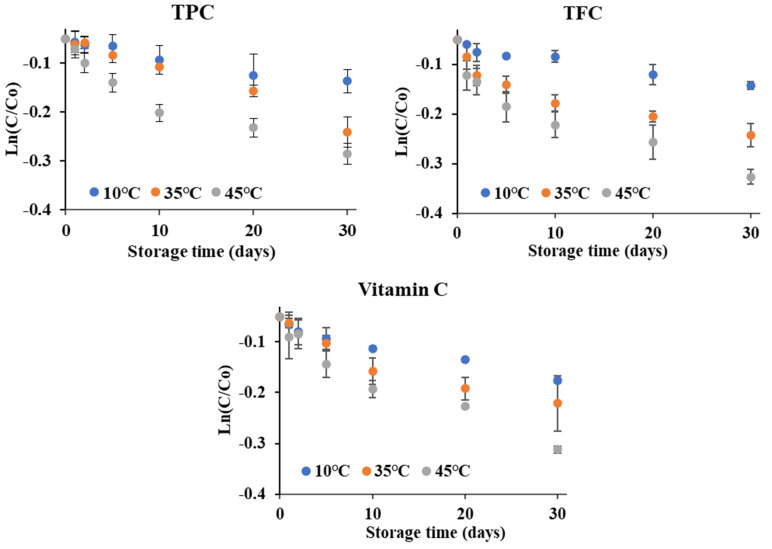
First order degradation plots for the TPC, TFC and Vitamin C content in encapsulated acerola powder under different storage temperatures.

**Figure 8:**
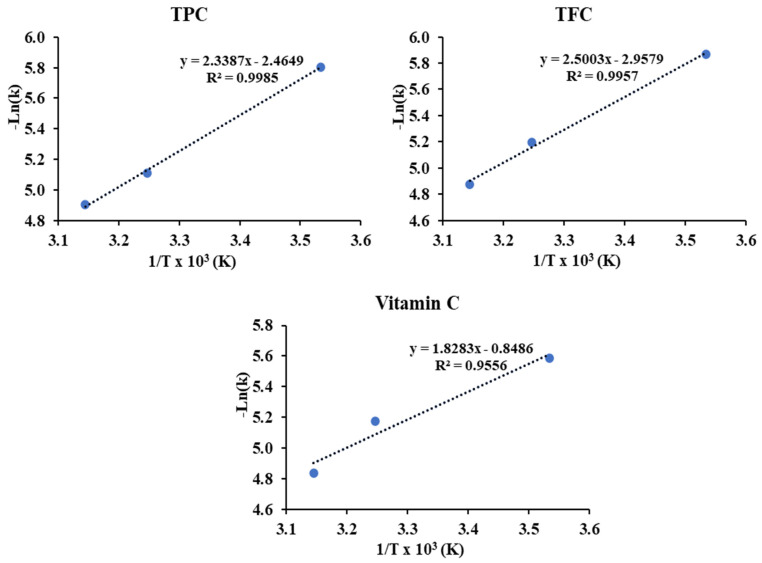
Arrhenius plots for the degradations of TPC, TFC, and Vitamin C in encapsulated acerola powder under different storage temperatures.

The first order degradation diagram, kinetic model parameters and Arrhenius diagram of TPC, TFC and vitamin C contents during 30 days of storage at three different temperature conditions are depicted in Figure 7, Table 8, and Figure 8, respectively.

As can be seen in Figure 7, the degradation of TPC, TFC, and vitamin C contents in the encapsulated acerola powder under different storage temperatures conforms to the first order reaction. This finding is consistent with the study conducted by Serea et al. [41] on the red grape skin. Table 8 illustrates the kinetic parameters of TPC, TFC, and Vitamin C in the encapsulated acerola powder. It is evident that similar degradation coefficients (k) indicate that the effects of storage temperature on TPC, TFC, and Vitamin C contents are nearly identical. Furthermore, this demonstrates that the degradation of the investigated parmeters occurs more prominently at higher storage temperatures and longer storage times, with a significantly lower k value at 10 °C (approximately half), compared to the k value at 40 °C. This aligns with research findings suggesting that the TPC in sumac sap has a decomposition coefficient eight times higher at a storage temperature of 55 °C than when stored at 2 °C [42]. In addition, the half-life (Table 8) exhibits a similar trend, with shorter durations at higher temperatures, as elevated temperatures can promote oxidation and structural changes in molecules, leading them to denaturation [39].

Furthermore, the activation energies of TPC and TFC, derived from the slopes of the Arrhenius plot shown in Figure 8, were found to be similar (4.65 kcal/mol and 4.97 kcal/mol, respectively), yet notably higher than the activation energy of Vitamin C (3.63 kcal/mol). This observation suggests that the temperature exerts a comparable effect on TPC and TFC, while the temperature-induced degradation of Vitamin C appears to be more pronounced. Additionally, the stability of the powder is notably higher when stored at 10 °C, with a discernible decline observed as storage temperature increases. 

Some studies have suggested that the presence of oil can degrade the quality and shelf life of powder. due to factors such as agglomeration or oil oxidation, leading to the formation of unexpected compounds and off-flavors [43]. However, this effect can be mitigated by various methods, such as adding antioxidants [44] or storing the product at low temperatures, anaerobically, and away from sunlight. In this study, the emulsifier Tween 80 is believed to possess antioxidant properties [45,46], which can enhance the oxidative stability of the encapsulated oil, thereby helping to maintain the quality and functionality of the oil over time. Moreover, no significant visual changes in the powder were observed during storage. Nonetheless, for optimal preservation and extended shelf life, it is recommended to store and vacuum seal the powder at low temperatures.

### 3.4. Effect of Humidity Conditions

**Figure 9:**
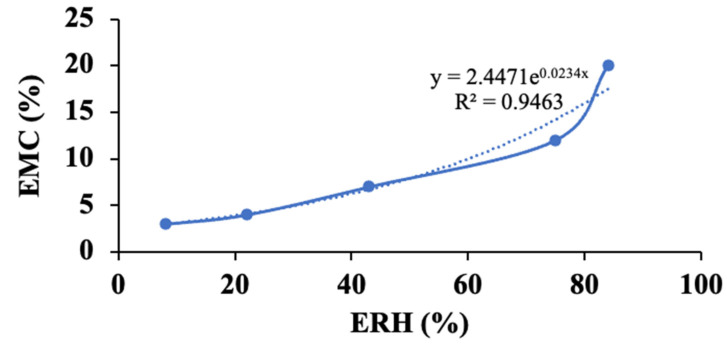
Effect of ERH on EMC of encapsulated powder.

The impact of air humidity on spray-dried acerola powder was investigated at 30 °C under five distinct humidity levels for a duration of 20 days until reaching weight equilibrium. Figure 9 illustrates the graphical relationship between the equilibrium moisture content (EMC, %) and the equilibrium relative humidity (ERH, %) at 30 °C, represented by a sigmoid curve. This curve offers valuable insights into the hygroscopic characteristics of the food product, enabling predictions regarding the influence of relative humidity variations on moisture content. Such predictions are crucial for quality control, storage and preservation practices [47].

In general, the EMC of the powder tends to rise with an increase in ERH. Within an ERH range below 40%, the moisture content and water activity of the powder remain relatively low, suggesting minimal free water content within the powder. Consequently, powder under such conditions exhibits stability in morphology and retains its physical, chemical, and biological properties. On the contrary, ERH levels exceeding 40% indicate accelerated water loss and a rapid decline in powder quality due to physical, chemical, and biological reactions. Therefore, it is recommended to store the powder in environmental conditions where the ERH remains below 40%, to maintain its quality over time.

The monolayer moisture content (M_o_) plays a crucial role in maintaining the quality and prolonging the shelf life of food products. It represents the minimum moisture content necessary to ensure storage stability for food and agricultural products [47]. Food products exhibit the highest stability when their moisture content or water activity matches the monolayer layer, and they become increasingly unstable when moisture levels deviate from this point. When humidity drops below the M_o_ value, physical, chemical, and biological reactions within food product slow down. Conversely, higher humidity levels can accelerate browning reactions, as well as enzymatic and microbial activities. Therefore, to maximize shelf life, the moisture content of dried product should ideally be kept below or approximately equal to the M_o_ value.

**Figure 10:**
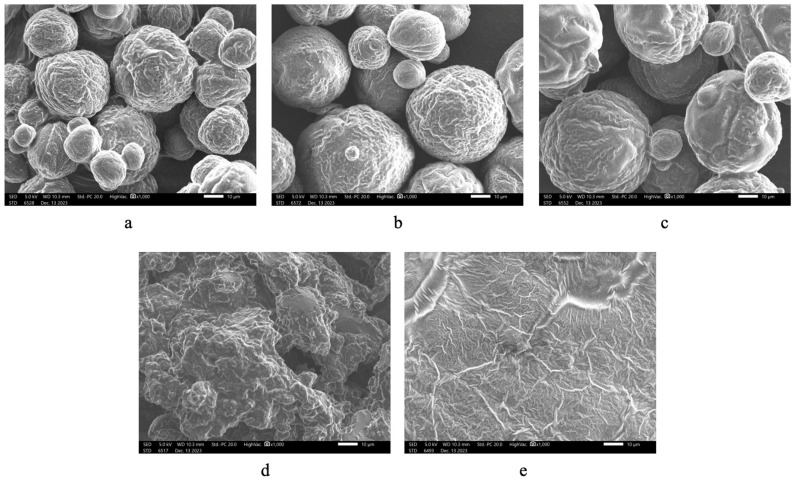
Powder particle morphology after reaching moisture equilibrium under five different air humidity conditions under 1000× magnification: (**a**) 8%, (**b**) 22% (**c**) 43%, (**d**) 75%, and (**e**) 84%.

Using the BET equation, the M_o_ value of the powder at 30 °C was predicted to be 4.01% (R^2^ = 0.983). This value aligns with the typical safe moisture content of most dried food products, which is approximately 4%. Furthermore, Figure 10 illustrates that the morphology of powder particles under EMC conditions close to or slightly lower than the M_o_ value (ERH less than 75%) exhibited similar characteristics (Figure 10a–c). However, powder particles subjected to ERH conditions at or higher than 75% (Figure 10d,e) displayed swelling and adhesion phenomena. At elevated ERH levels, the powder adsorbs more moisture from the surrounding environment, leading to the condensation of moisture on the surface of powder particles. This condensation forms liquid bridges between particles, causing them to adhere together [48]. In addition, the moisture content of encapsulated powder spray dried at optimal inlet and outlet temperatures is approximately 3.21%, which is lower than the M_o_ value. This indicates that the powder possesses the capability for prolonged preservation and utilization. 

**Figure 11:**
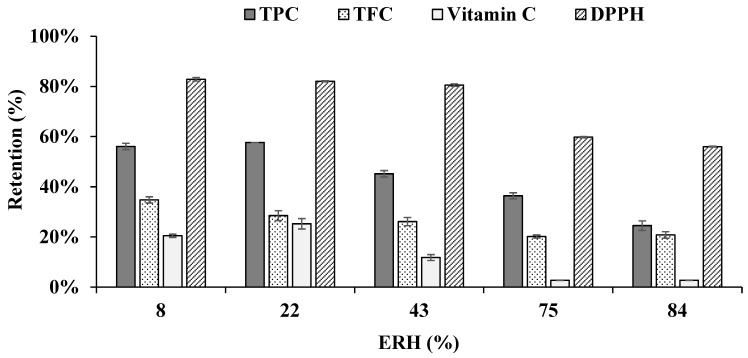
Effect of equilibrium relative humidity (ERH) on the retention of TPC, TFC, Vitamin C, and DPPH-free radical scavenging ability of acerola encapsulated powder.

The content of bioactive compounds in encapsulated powder was strongly affected by moisture exchange with the environment. Figure 11 shows that the retention of TPC, TFC, Vitamin C, and DPPH-free radical scavenging ability of encapsulated powder decreased with increasing relative air humidity. This observation can be attributed to the relationship between relative humidity and oxidation. High ERH can elevate moisture content, consequently accelerating the oxidation process [49]. This acceleration leads to a reduction in Vitamin C content, causing phenolic compounds to leach out or degrade, thereby resulting in a decrease in TPC and TFC [50]. Additionally, high ERH can impact the stability of the antioxidants being measured, further contributing to a decrease in DPPH radical scavenging activity [51]. Therefore, to maximize the storage time at 30 °C, the EMC of spray-dried acerola powder should ideally be maintained below 4.01%.

The apparent robust stability of bioactive compounds within encapsulated powder products during storage is highly advantageous. However, another critical consideration pertains to the release of polyphenolic compounds and Vitamin C from the GA–MD matrix, along with the preservation of their antioxidant activity under gastric and intestinal conditions. The behavior of the encapsulation system under digestive conditions ultimately dictates the bioavailability of bioactive compounds and the potential utility of encapsulated acerola powder in meeting recommended daily intake levels for these compounds. Additionally, processing factors, such as emulsification and spray drying conditions, may modestly influence bioavailability, albeit to a lesser extent. Hence, further research is warranted to comprehensively elucidate the bioavailability of encapsulated powder.

As previously noted, RDI levels for Vitamin C are established at 75 mg and 90 mg per day. Table 6 illustrates that the Vitamin C content (123.83 mg/100g) remains substantially higher than the recommended levels, even after storage at low temperatures for over 8 months. The findings of this study affirm the sustained high levels of Vitamin C in the encapsulated product during storage, thereby positioning the product as a commendable source of Vitamin C. However, as of now, there is no official establishment for an RDI of total polyphenols [52,53], although an approximate mean intake of about 900 mg/day has been reported [52]. Given that the prevention of numerous chronic diseases is linked to consuming approximate RDI level of polyphenols, the encapsulated arocela powder containing polyphenols and Vitamin C should be considered as a nutrient supplement for health benefits.

## 4. Conclusions

The optimized inlet and outlet temperatures of the spray drying process, determined via the RSM–CCD model, were 157 °C and 91 °C, respectively. Quadratic equations were established to predict the effects of these temperatures on moisture content (R^2^ = 0.93), process yield (R^2^ = 0.91), the EY_TPC_ (R^2^ = 0.97), EY_TFC_ (R^2^ = 0.95) and EY_vitamin C_ (R^2^ = 0.88). Notably, spray drying temperature did not significantly affect bulk density.

Bioactive compounds in encapsulated acerola powder exhibited good release characteristics in solvents simulating hydrophilic food environments. Rapid release occurred within the first 10 min, with enhanced release observed in acidic environments. Additionally, the release mechanism of optimal encapsulated powder was predicted by the Korsmeyer–Peppas model and diffusion by Fick’s law.

Powder stability, as influenced by morphology and encapsulation performance of bioactive compounds, is impacted by air humidity due to water adsorption. Appropriate relative humidity for long-term powder storage should maintain below 40%, ensuring an equilibrium moisture content below the M_o_ value of 4.01% predicted by the BET model. Moreover, encapsulated acerola powder dried under optimal conditions demonstrated greater stability when stored at 10 °C, compared to 30 °C or 40 °C, suggesting cold storage for long-term use.

In summary, the encapsulated acerola W/O/W emulsion powder demonstrates stability when stored under low temperatures and controlled air humidity conditions. The high quality of the encapsulated powder renders it suitable for use as a nutrient supplement, owing to its significant content of polyphenols and Vitamin C, along with its retained antioxidant activity.

## Figures and Tables

**Table 1:** Inlet (X_1_) and outlet (X_2_) temperature levels for the CCD–RSM model.

Coded Variable Levels	Inlet Temperature (X_1_, °C)	Outlet Temperature (X_2_, °C)	Feed Flow Rate (mL/h)
+1.414	162	97	378
+1	160	95	420
0	155	90	474
−1	150	85	498
−1.414	148	83	540

**Table 2:** Bulk densities of the encapsulated powder obtained with the different inlet and outlet temperatures.

X_1_ (°C)	X_2_ (°C)	Bulk Density (g/mL)
150	85	0.21 ^a^ ± 0.01
160	85	0.21 ^a^ ± 0.01
150	95	0.23 ^a^ ± 0.01
160	95	0.21 ^a^ ± 0.01
148	90	0.23 ^a^ ± 0.01
162	90	0.22 ^a^ ± 0.01
155	83	0.22 ^a^ ± 0.01
155	97	0.21 ^a^ ± 0.01
155	90	0.23 ^a^ ± 0.01
155	90	0.23 ^a^ ± 0.01
155	90	0.23 ^a^ ± 0.01

Values in a column sharing a superscript letter are not significantly different from each other (*p* > 0.05).

**Table 3:** Experimental (Exp.) and predicted (Pred.) data of the response variables of the encapsulated powders obtained from the CCD–RSM model.

Pattern *	X_1_ (°C)	X_2_ (°C)	MC (%)		Process Yield (%)		EY_TPC_ (%)		EY_TFC_ (%)		EY_vitamin C_ (%)	
			Exp.	Pred.	Exp.	Pred.	Exp.	Pred.	Exp.	Pred.	Exp.	Pred.
--	150	85	3.68	3.72	80.94	78.05	43.75	45.13	29.18	29.89	24.59	23.34
+-	160	85	3.37	3.47	92.63	91.02	49.93	50.97	26.73	29.02	23.86	23.11
-+	150	95	3.48	3.39	80.57	80.89	42.66	43.69	25.65	23.92	22.82	22.90
++	160	95	2.97	2.94	84.6	86.20	53.4	54.09	33.48	33.93	23.74	24.31
a0	148	90	3.35	3.39	74.31	75.86	40.73	39.46	21.66	21.99	20.83	21.52
A0	162	90	2.95	2.90	89.05	88.79	51.74	50.94	31.35	28.45	22.36	22.35
0a	155	83	4.01	3.92	83.55	86.46	52.43	51.15	36.94	33.54	23.36	24.63
0A	155	97	3.23	3.31	86.69	85.06	53.12	52.33	32.55	32.79	25.77	25.17
00	155	90	2.98	3.12	89.23	90.31	59.77	61.10	36.72	37.40	28.36	27.50
00	155	90	3.12	3.12	91.32	90.31	60.77	61.10	37.75	37.40	27.47	27.50
00	155	90	3.26	3.12	90.37	90.31	62.77	61.10	37.72	37.40	26.68	27.50

* Experiments were conducted in a random order.

**Table 4:** Coded second order regression coefficients for moisture content, process yield, the EY of TPC, TFC, and Vitamin C content.

Regression Coefficient ^a^	MC (%)	Process Yield (%)	EY_TPC_ (%)	EY_TFC_ (%)	EY_vitamin C_ (%)
a_o_	3.12	90.31	61.10	37.40	27.50
Linear					
a_1_	−0.17 *	4.57 **	4.06 **	2.28 *	0.29
a_2_	−0.21 **	−0.49	0.42	−0.27	0.19
Quadratic					
a_1_a_1_	0.013	−3.99 *	−7.95 ***	−6.09 **	−2.79 **
a_2_a_2_	0.25 **	−2.27	−4.68 **	−2.12 *	−1.30 *
Interaction					
a_1_a_2_	−0.05	−1.91	1.14	2.72 *	0.41
R^2^	0.93	0.91	0.97	0.95	0.88
*p*-value of lack of fit	0.64	0.11	0.46	0.07	0.32

^a^ a_o_ is a constant, a_i_, a_ii,_ and a_ij_ are the linear, quadratic, and interactive coefficients of the surface model, respectively. *: *p* < 0.05; **: *p* < 0.01; ***: *p* < 0.001.

**Table 5:** Validation of the CCD–RSM model using the optimized inlet (157 °C) and outlet (91 °C) temperatures.

	MC (%)	Process Yield (%)	EY_TPC_ (%)	EY_TFC_ (%)	EY_vitamin C_ (%)
Predicted values	3.02	91.15	61.44	37.42	27.19
Experimental values	3.21 ± 0.21	90.23 ± 1.05	58.06 ± 0.01	35.19 ± 0.03	26.42 ± 0.02

**Table 6:** HPLC analysis results of powders spray-dried at optimal temperatures.

Compounds	Content
Vitamin C (mg/100 g d.w.)	123.83 ± 11.02
Gallic acid (mg/100 g d.w.)	143.91 ± 2.24
Catechin (mg/100 g d.w.)	89.09 ± 3.49
Epicatechin (µg/100 g d.w.)	83.06 ± 0.03

**Table 7:** Kinetics and release mechanism of Vitamin C, TPC, TFC, and DPPH under different conditions.

	Release Media	Korsmeyer-Peppas Model			Peppas-Sahlin Model				Higuchi Model		Mechanism
		K_M_	n	R^2^	K_1_	K_2_	n	R^2^	K_H_	R^2^	
Vitamin C	Water	4.975	0.159	0.990	4.819	0.172	0.151	0.990	1.342	0.847	Fick
	Ethanol	4.648	0.169	0.983	3.363	1.327	0.125	0.984	1.304	0.865	Fick
	Acetic acid	4.803	0.170	0.992	4.658	0.166	0.161	0.992	1.343	0.851	Fick
TPC	Water	0.138	0.064	0.995	0.013	0.125	0.033	0.995	0.026	0.633	Fick
	Ethanol	0.123	0.071	0.996	0.085	0.039	0.052	0.996	0.024	0.653	Fick
	Acetic acid	0.135	0.077	0.992	0.087	0.048	0.056	0.992	0.027	0.674	Fick
TFC	Water	0.103	0.141	0.996	0.000	0.103	0.071	0.996	0.026	0.814	Fick
	Ethanol	0.102	0.144	0.989	0.089	0.014	0.123	0.989	0.026	0.834	Fick
	Acetic acid	0.105	0.148	0.988	0.000	0.120	0.056	0.981	0.027	0.807	Fick
DPPH	Water	60.151	0.066	0.991	60.141	0.010	0.066	0.991	11.575	0.637	Fick
	Ethanol	64.931	0.033	0.998	64.931	0.000	0.033	0.998	11.111	0.531	Fick
	Acetic acid	62.246	0.044	0.999	61.956	0.038	0.045	0.999	11.127	0.593	Fick

**Table 8:** Degradation kinetics of TPC, TFC and Vitamin C in the encapsulated acerola powder.

Storage Temperature	TPC			TFC			Vitamin C		
	k (day^−1^)	Half-Life (Days)	R^2^	k (day^−1^)	Half-Life (Days)	R^2^	k (day^−1^)	Half-Life (Days)	R^2^
10 °C	0.0030	230	0.96	0.0028	245	0.95	0.0037	185	0.95
35 °C	0.0060	115	0.99	0.0055	125	0.86	0.0057	123	0.93
45 °C	0.0074	93	0.91	0.0076	91	0.87	0.0079	87	0.94

k: Apparent degradation rate constant; R^2^: Correlation coefficient.

## Data Availability

The original contributions presented in the study are included in the article, further inquiries can be directed to the corresponding author.
